# Prediction of the Transition From Subexponential to the Exponential Transmission of SARS-CoV-2 in Chennai, India: Epidemic Nowcasting

**DOI:** 10.2196/21152

**Published:** 2020-09-18

**Authors:** Kamalanand Krishnamurthy, Bakiya Ambikapathy, Ashwani Kumar, Lourduraj De Britto

**Affiliations:** 1 Department of Instrumentation Engineering Madras Institute of Technology Campus Anna University Chennai India; 2 Vector Control Research Centre Indian Council for Medical Research Puducherry India

**Keywords:** COVID-19, epidemic, mathematical modeling, probabilistic models, public transport, exponential transmission

## Abstract

**Background:**

Several countries adopted lockdown to slowdown the exponential transmission of the coronavirus disease (COVID-19) epidemic. Disease transmission models and the epidemic forecasts at the national level steer the policy to implement appropriate intervention strategies and budgeting. However, it is critical to design a data-driven reliable model for nowcasting for smaller populations, in particular metro cities.

**Objective:**

The aim of this study is to analyze the transition of the epidemic from subexponential to exponential transmission in the Chennai metro zone and to analyze the probability of severe acute respiratory syndrome coronavirus 2 (SARS-CoV-2) secondary infections while availing the public transport systems in the city.

**Methods:**

A single geographical zone “Chennai-Metro-Merge” was constructed by combining Chennai District with three bordering districts. Subexponential and exponential models were developed to analyze and predict the progression of the COVID-19 epidemic. Probabilistic models were applied to assess the probability of secondary infections while availing public transport after the release of the lockdown.

**Results:**

The model predicted that transition from subexponential to exponential transmission occurs around the eighth week after the reporting of a cluster of cases. The probability of secondary infections with a single index case in an enclosure of the city bus, the suburban train general coach, and the ladies coach was found to be 0.192, 0.074, and 0.114, respectively.

**Conclusions:**

Nowcasting at the early stage of the epidemic predicts the probable time point of the exponential transmission and alerts the public health system. After the lockdown release, public transportation will be the major source of SARS-CoV-2 transmission in metro cities, and appropriate strategies based on nowcasting are needed.

## Introduction

Severe acute respiratory syndrome coronavirus 2 (SARS-CoV-2), or the coronavirus disease (COVID-19) emerged in Wuhan, China and has quickly spread to most of the countries around the world. As of May 10, 2020, 3,917,366 COVID-19 cases and 274,361 related deaths were reported worldwide. At the same time, the Ministry of Health and Family Welfare, India reported 62,939 confirmed cases and 2109 deaths in India. India has 28 states and 8 union territories, out of which 26 states and 7 union territories have reported COVID-19 cases. However, a large proportion of the cases were reported from the 4 states Maharashtra, Tamil Nadu, Gujarat, and Delhi. The case-fatality rate in India remains low as compared to the global rate (7.0% vs 3.35%) [1].

The estimated population of the Tamil Nadu State for the year 2020 is 82.2 million and is the seventh most populated state in India. It has 37 districts and Chennai is the largest and most populated city in Tamil Nadu, and, based on the nationwide census in 2011, the projected total population of Chennai District is around 4,935,550 [2]. The whole geographical zone of Chennai District is well connected through two major public transport systems: Metropolitan Transport Corporation (MTC) and Chennai Suburban Railways. These transports are also extended to the three bordering districts, namely, Kanchipuram, Chengalpattu, and Thiruvallur. The Department of Health and Family Welfare of Tamil Nadu reported a total of 3839 COVID-19 cases in Chennai, 267 cases in Chengalpattu, 122 cases in Kanchipuram, and 337 cases in Thiruvallur, as of May 10, 2020. The maximum number of infected cases were registered in Chennai [3], and the first SARS-CoV-2 infection was reported in Kanchepurram District on March 7, 2020.

**Figure 1:**
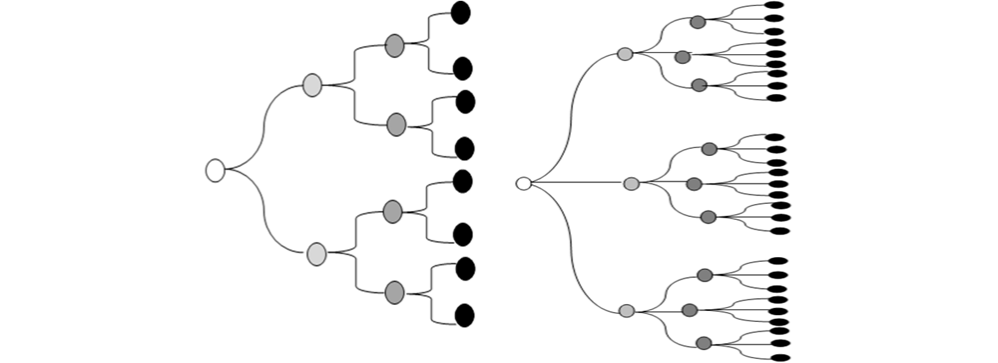
Increase in the number of cases over subsequent generations of the infection for (a) a reproductive number of 2 and (b) a reproductive number of 3.

Public transportation such as trains and buses is an essential service with specific route systems. The Chennai suburban railway consists of two major networks: Chennai Suburban Railway Network and Mass Rapid Transport System; as of 2015-2016, it carried about 1.17 million passengers every day [4]. The MTC operates 3233 services and carries about 3.3 million passengers per day [5]. In the current COVID-19 pandemic situation, commuting in public transport is associated with two major risks: asymptomatic passengers play a major role in SARS-CoV-2 transmission through aerosol particles and indirect transmission from symptomatic passengers may occur through fomites. Furthermore, public transport employees are at a higher risk of infection for long hours with multiple sources of exposure [6]. Even an increase in the reproductive number (R_0_) from a value of 2 to 3 leads to a significant amplification in the number of infected cases over subsequent generations, as shown in Figure 1.

Mathematical modeling plays an important role for predicting, assessing, and controlling potential outbreaks for infectious diseases such as H1N1 [7], severe acute respiratory syndrome (SARS) [8], Middle East respiratory syndrome (MERS) [9], and Ebola [10]. At present, several researchers have used mathematical modeling to predict the SARS-CoV-2 pandemic using model structures such as the susceptible-infected-recovered model, the exponential model, and the susceptible-exposed-infected-removed model [11-13]. The early epidemic growth can be well-drawn using subexponential and exponential models. Such models are highly appropriate when there is a major uncertainty regarding the epidemiology of a novel infectious disease, for which the transmission pathways are not completely known. In such cases, subexponential and exponential models serve as reasonable tools for analyzing the progression of the early epidemic and for short-time prediction of the infected cases in the near future [14].

Recently, modeling approaches have been used for analysis of the transmission of COVID-19 infection with travel interventions [15]. Anzai et al [16] have investigated the impact of travel interventions inside and outside of China during the COVID-19 pandemic and concluded that travel intervention during the COVID-19 pandemic resulted in less cases. However, a significant number of infected individuals with mild or no symptoms are likely to pass through border control if travel interventions are not imposed properly.

When India had gone through 5 weeks of continuous lockdown during the last week of April 2020, there were 33,050 confirmed cases and 1074 deaths [17]. An overview of the case distribution indicated that there were more from urban clustering, in particular, with the three major metro cities Mumbai, Delhi, and Chennai. Therefore, nowcasting was proposed for the COVID-19 epidemic in the Chennai metro zone using different predictive mathematical models to generate an evidence for focused public health interventions in metro zones. In support of this, the probability of infection and the related secondary infections due to the COVID-19 infected population in public transport systems such as buses and train coaches is analyzed using probabilistic models.

## Methods

### Study Site

India reported more than 100,000 cases of COVID-19 as of May 18, 2020, even after consecutive lockdown for a period of 55 days. Though the epidemic was slowed down as expected, 3 states contributed more than 58% of the total cases in the country and in each State more than 60% of the cases were reported from the respective capital cities Mumbai, Chennai, and Ahmadabad. Therefore, containment of the SARS-CoV-2 transmission in these three cities is critical in favorably modifying the transmission in India. These 3 cities share the same characteristics in terms of population structure, density, and movement of the people toward these cities for employment.

**Figure 2:**
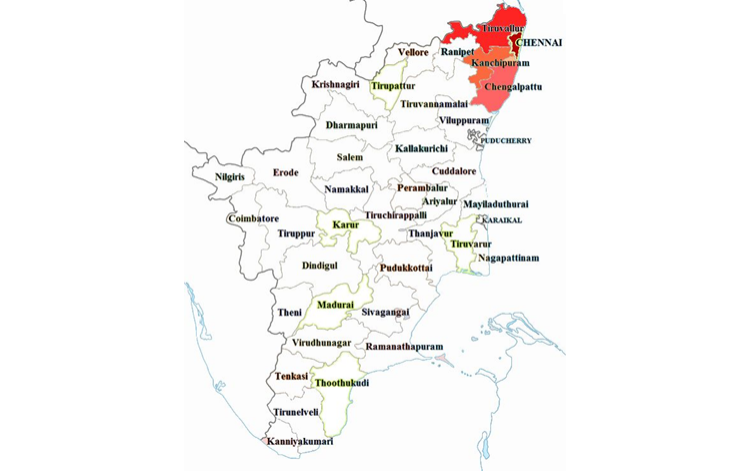
Constructed study site “Chennai-Metro-Merge,” combining Chennai District with the three bordering districts Chengalpattu, Kanchepuram, and Thiruvallur. The estimated total population of the constructed single geographical zone by 2020 is 15,208,505.

Chennai is a metropolitan city surrounded by three other districts Kancheepuram, Thiruvallur, and Chengalpattu. Based on the connectivity of the three transport systems, widespread locations of the educational institutes, and the movement of the population from these three districts into every part of Chennai, we felt it appropriate to predict the SARS-CoV-2 transmission considering all four districts as a single unit. In this study, we construct a single geographical zone “*Chennai-Metro-Merge*” by combining Chennai District with the bordering three districts for the development of a predictive model (Figure 2).

### Modeling of the COVID-19 Epidemic in Four Districts of Tamil Nadu Using Subexponential and Exponential Models

In this study, the total reported COVID-19 cases in the constructed geographical zone *Chennai-Metro-Merge* (Figure 2) were considered for the development of a predictive model. The number of infected cases from March 7, 2020, to April 30, 2020, was adopted from the open-source data provided by the Department of Health and Family Welfare, Government of Tamil Nadu [18] and was used for modeling the short-term progression (nowcasting) of the epidemic in these four districts, considered in a single geographical boundary since these four districts are well connected by roadways and suburban train services for public movement and the movement of materials. The nowcasting was further extended up to June 30, 2020, by adopting the reported cases from May 16 to June 10, 2020.

Two different models were considered for the study. First, an exponential model of the form:


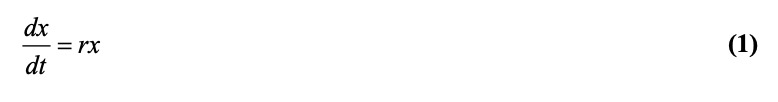


The solution of equation 1 is given as:

*x*(*t*) = *x*(0)*e^rt^*                 ** (2)**

Second, a subexponential model of the form:


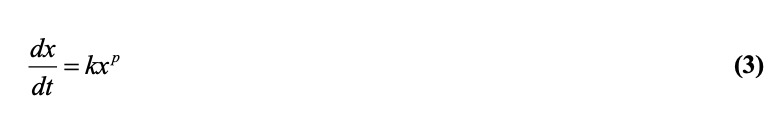


with solution:


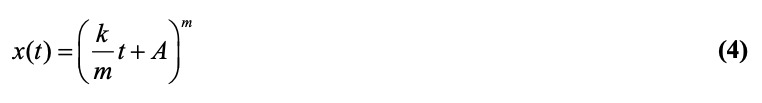


where, *x*(*t*) is the number of infected cases at time *t*, 
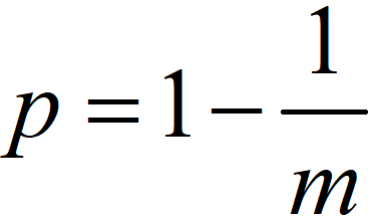
, and 
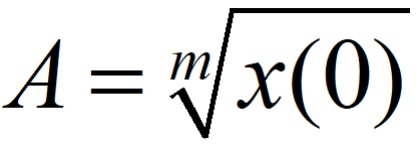
 [17].

This study uses the subexponential and the exponential models to estimate the date of transition of the epidemic, and in the field of epidemiology, these models are well suited for the study of the early epidemic growth [19]. Using the reported cases, the parameters of both the considered models were estimated using the minimization of the objective function given by:


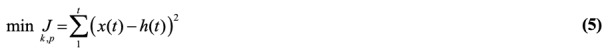


where, *x*(*t*) is the model output, and *h*(*t*) is the reported infections at *t*^th^ day. The optimization problem was solved using the MATLAB (MathWorks) programming software. The subexponential and exponential models were analyzed, and a technique for the prediction of the onset date of exponential transmission was identified. Furthermore, the developed model was simulated to approximately predict and analyze the future COVID-19 infections in these four districts.

### Analysis of COVID-19 Transmission due to Public Transport in the Considered Districts of Tamil Nadu

In an enclosed environment, the number of secondary infections (*R_A_*) arising due to the introduction of infectious cases into the susceptible population in an enclosed environment is given by:

*R_A_* = (*N* – *I*)*P*                  ** (6)**

where, *N* is the total population inside the enclosed environment such as buses or train compartments, *I* is the number of infected individuals inside the same enclosed environment, and *P* is the probability of infection. Equation 6 was used to analyze the transmission of COVID-19 in buses and train compartments when the lockdown is released and the public transportation is resumed in Tamil Nadu. The buses in Tamil Nadu are to be operated with 50% capacity on the immediate release of the lockdown.

The probability of infection *P* is given by:


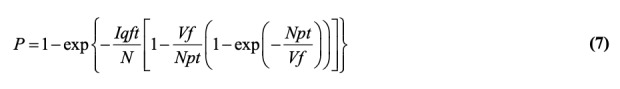


where, *N* is the number of individuals in the bus or train compartment, *V* is the volume of shared air space in *m*^3^, *t* is the total exposure time in hours, *p* is the breathing rate in *m*^3^/hour, *f* is the fraction of indoor air exhaled by the infected people, *q* is the quantum generation rate, and *I* is the initial number of infected people. The values of *q*, *f*, and *p* were adopted from [20]. The volume of the single train coach was considered from the literature [21]. Further, the maximum initially infected in the bus, train coach, and the ladies’ compartment in the train was assumed as 3, 4, and 3, respectively. This assumption is based on the volume of the bus and the train, the number of passengers, and the commuter density in the bus stops and railway stations.

## Results

**Figure 3:**
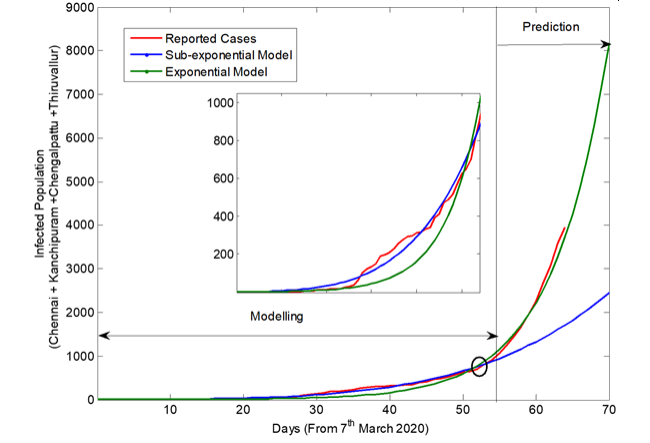
The reported number of coronavirus disease cases (includes effect of intervention), and the output of the subexponential and the exponential models, shown as a function of time.

Figure 3 shows the exponential and the subexponential models fitted to the reported number of infections in the four considered districts as a function of time in days. The data available from March 7, 2020, to April 29, 2020, was used to generate the models, and the predictions are further presented up to May 15, 2020. It was observed that, during the early stage of the epidemic, the subexponential model best describes the progression of the infected cases. However, after a particular point of time, infected cases are closely tracking the curve described by the exponential model. The week in which the transition from the subexponential to the exponential progression begins is an important marker of the change in the course of the epidemic, as described in Figure 3. Both the developed models were simulated to predict the future number of COVID-19 cases, and the resulting curves were compared with the actual reported cases. It is seen that there was no uniform pattern in the day-to-day reporting of the cases. Therefore, initially, the progression trend of the reported number of infections is close to the predictions made by the exponential phase and in short period to the subexponential phase. However, the merging and the transition from the subexponential to exponential phase was clearly visible at a particular time point.

**Figure 4:**
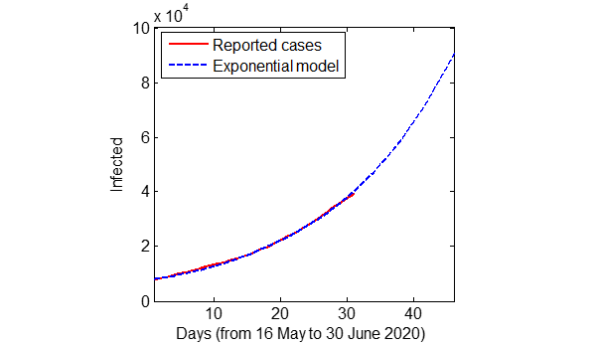
The total coronavirus disease cases in the four considered districts of Tamil Nadu predicted using the updated exponential model and the actual reported cases.

The exponential model was further updated using the data available from May 16 to June 10, 2020, and the model was used to further predict the future number of cases up to June 30, 2020, in the considered geographical boundary. The updated exponential model output and the reported cases are shown in Figure 4.

**Figure 5:**
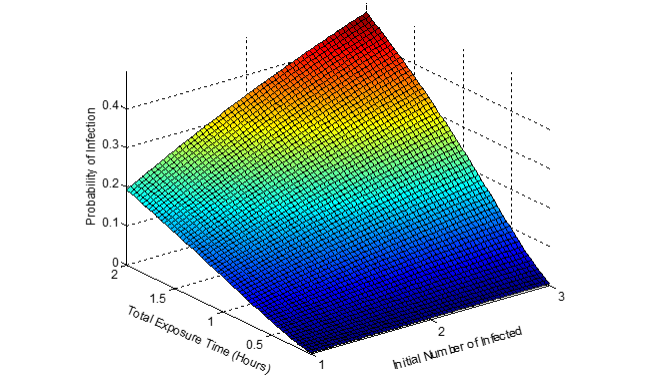
The probability of infection in a public bus with 20 passengers shown as a function of the total exposure time and the initial number of infected.

**Figure 6:**
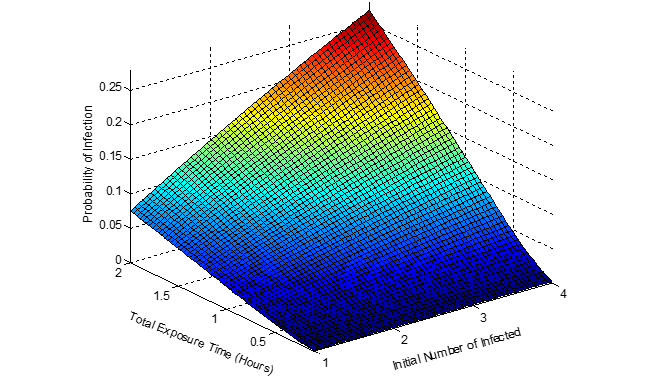
The probability of infection in a single train coach with 54 passengers shown as a function of the total exposure time and the initial number of infected.

**Figure 7:**
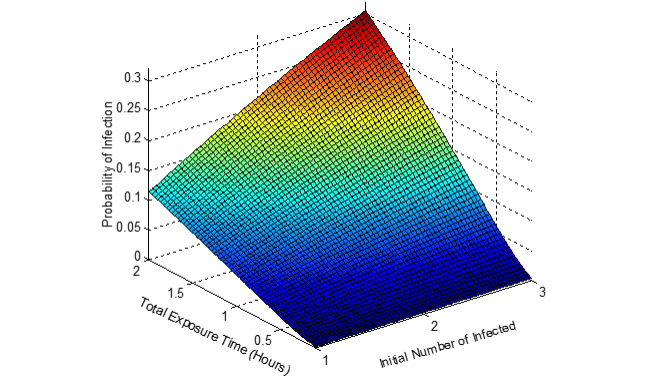
The probability of infection in a single train coach (ladies compartment) with 36 passengers shown as a function of the total exposure time and the initial number of infected.

**Figure 8:**
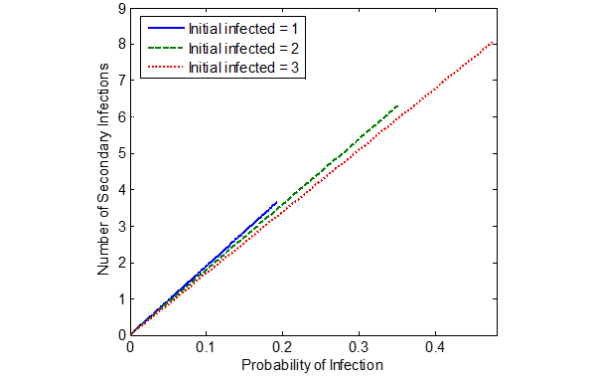
The number of secondary infections in the bus due to the introduction of infected individuals into the susceptible population (total population of N = S + I = 20), shown as a function of the estimated probability of infection.

**Figure 9:**
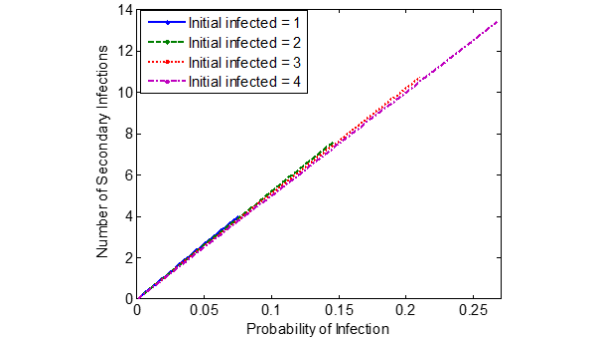
The number of secondary infections in the train compartment due to the introduction of infected individuals into the susceptible population (total population of N = S + I = 54), shown as a function of the estimated probability of infection.

**Figure 10:**
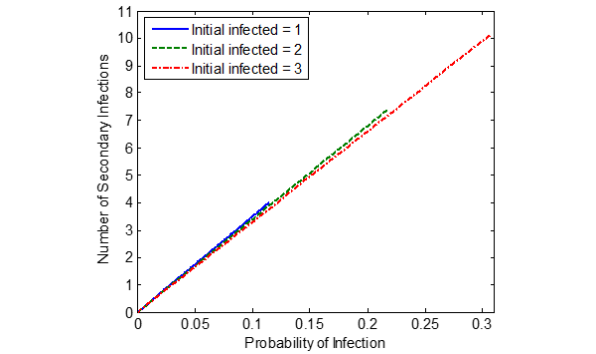
The number of secondary infections in the train coach (ladies compartment) due to the introduction of infected individuals into the susceptible population (total population of N = S + I = 36), shown as a function of the estimated probability of infection.

Figures 5-7 show the probability of infection as a function of both the travelling time (exposure time) and the number of infected individuals travelling in the bus, a single train coach, and a single train coach (ladies compartment) with a total of 20 passengers, 54 passengers, and 36 passengers, respectively. It is seen that the increase in the initial number of infected individuals and the increase in the exposure time leads to an increased probability of infection of the susceptible. In the bus, for an exposure time of 2 hours and with 3 initial infected individuals, the probability of infection is around 0.4741 (47.41%). Furthermore, the number of secondary infections arising due to the infected individuals travelling in the bus, single train compartment, and a single train coach (ladies compartment) is shown in Figures 8-10, respectively, as a function of the probability of infection and for various numbers of initially infected individuals. The results demonstrated that the operation of the train coaches at a reduced capacity of 50%, provides a maximum probability of infection of 0.2674. Furthermore, the maximum probability of infection in a single train coach (ladies compartment) was found to be 0.3061.

## Discussion

### Principal Findings

In this study, we constructed a mathematical model based on the reported cases from March 7, 2020, to April 29, 2020, to analyze the transition of the COVID-19 epidemic from the subexponential to the exponential stage in the combined Chennai metro-merge. Furthermore, the reported cases from May 16 to June 10, 2020, were used to update the exponential model to nowcast the progression of the epidemic up to June 30, 2020. Currently, five metro cities in India and several cities in South East Asian Region are facing a similar SARS-CoV-2 epidemic. The results of the modeling indicated that the transmission in all the four districts exhibited exponential transmission from the third or fourth week of the first reported case in each district. However, the number of predicted cases for this period was considerably less, and there was an opportunity until the eighth week (ie, the first week of May 2020) to favorably contain the epidemic and reverse to the subexponential transmission. On the other hand, the Government of Tamil Nadu proposed resuming both the bus and train services initially for the officials followed by the public in a phased manner. In public health, as well as the individual perspective, it is desirable to assess the risk of acquiring the SARS-CoV-2 infection while travelling for a considerable period of time in an enclosed environment. We used a probabilistic model and observed that the probability of acquiring the infection in the event of a single index case in the closed environment is lower in suburban train travel with a restricted occupancy of 50% as compared to the bus travel with the same proportion of occupancy (0.19 vs 0.07). The results also indicate that during the suburban train travel the probability of infection is higher in the ladies’ compartment as compared to the open compartment for an exposure time of 2 hours and when a single infected case is introduced (0.11 vs 0.07).

For the predictions to be reliable, the model parameters were estimated with the reported values using the optimization technique of the minimization of the sum square error between the model outputs and the reported values. In addition, standard probabilistic models were used to analyze the probability of infection in buses and trains, which are to be operated at reduced passenger loads after the release of the lockdown. Using the probability of infection due to the total exposure or travel time of the passengers and the initial number of infected individuals travelling in the bus or train coach, the numbers of possible secondary infections were estimated. The modes of SARS-CoV-2 transmission in an enclosed environment are droplet nuclei from the asymptomatic persons as well as the aerosol droplet, especially when the infected person sneezes or coughs during travel. It had been reported earlier in SARS-CoV-1 transmission that all the passengers infected during the flight travel were seated in close proximity to index cases [22]. Another investigator showed that the persistence of SARS viruses is longer compared to the influenza virus [23]. Therefore, there are likely to be a higher number of SARS-CoV-2 secondary infections during the bus and train travel compared to those reported for SARS-CoV-1 transmission [24]. Data-driven estimates in China during the early phase of the SARS-CoV-2 epidemic showed a highly significant association with train travel [25]. A comprehensive review by Perri et al [26] revealed that the massive rail connectivity to Wuhan in China favored the widespread transmission.

The SARS-CoV-2 pandemic has gone through several continents in a short span of 12 weeks, and the length of the epidemics in various countries indicate that there is likely to be a prolonged pandemic for a period of 18-24 months as observed in the Spanish flu pandemic in the early twentieth century. Based on the R_0_ during the initial phase of the epidemic in China, it is estimated that about 60% of the population will be infected if the epidemic is not mitigated [27]. Modeling studies suggest that to contain the epidemic before the exponential phase, about 70% of the contacts must be traced and quarantined [28]. Data from the earlier phase of the epidemics outside China indicate that nearly 80% of the infected remain asymptomatic or mildly symptomatic and resolve by self-healing [29].

Disease transmission models and the epidemic forecasts at national levels provide valuable information for the policy makers to implement appropriate intervention strategies in an appropriate time. However, it is critical to design data-driven reliable models for nowcasting and for smaller populations where clustering of transmission occurs. It is a routine practice among the public health specialists to rely on mechanistic epidemic models, and the major disadvantage with these models is that there is an underlying assumption of exponential transmission during the early phase of the epidemic itself [30,31] and, therefore, the predicted number of cases after 12 weeks or the final size of the epidemic is unusually high. Forecasts on the final size of the HIV and Ebola epidemics proved this phenomenon [32,33]. In our model, we considered both the subexponential and exponential transmission, and attempted to identify the time point at which there is a transition from subexponential to the exponential phase. The model predicted that the transition for the constructed geographical zone on Chennai-Metro-Merge falls at the eighth week of the epidemic. To avoid the unrealistic size of the epidemic for a small geographical area, we restricted the nowcasting approach to predict the number of cases for the next 6 weeks. In a nation-wide epidemic of SARS in large countries like India, there must be two levels of transmission control, one at the national level and the other at the state level. At both the levels, it is necessary to plan for the early forecast instead of identifying the magnitude of the epidemic as short-term; timely projections provide an opportunity for the type and intensity of interventions for the particular population [34] so that the epidemic is contained without causing any strain on public health infrastructure. It is important to know the size of the epidemic for the budget allocation and the mobilization of the public health infrastructure.

The major limitations of the study with reference to the predictions are that the data inputs for the study were based on the limited numbers of testing in the study districts and the limited period of predictions for only 6 weeks. In addition, when calculating the probability, we assumed the maximum possible number of initial infections in a single enclosure in a bus or train from the initial part of the journey as 3 and 4, respectively. Chennai metro services are always five times overcrowded during peak hours, and most of the enclosures are expected to be full if the restrictions on the occupancy are imposed during the initial phase of the release. With the current exponential trends, even with random contact, the passengers are likely to be exposed repeatedly during the point to point travel for a period of 2 hours.

At present, several countries are going through the early phase or the subexponential phase of the epidemic and have not yet reached the exponential phase; the methods, results, and experiences reported in this work are of high value in undertaking midcourse corrections in the implementation of the intervening strategies to contain the SARS-CoV-2 epidemic. The developed model in this study is simple and can be constructed easily in any software package using the reported infections over a period of time. Hence, this methodology can be adopted by public health specialists and epidemiologists to trace the current trend of the epidemic and to nowcast the progression of the epidemic at a small population level like in metro cities and districts.

### Conclusions

Though all the countries are well aware of the rapid response to the epidemics, each epidemic exhibits certain challenges. There are several challenges during the current COVID-19 epidemic globally and locally. India imposed lockdown as an intervention reasonably in advance as compared to other countries. However, this epidemic has shown categorically that lockdown alone is insufficient to contain the epidemic. Lockdown provides an opportunity for the symptomatic to surface out so that the contacts are traced, quarantined, and the severe forms of the diseases or complications are identified and treated. As shown by Keeling et al [28], it is essential to trace about 70% of the contacts to contain the epidemic spread. China succeeded in the COVID-19 epidemic control by strictly imposing lockdown, but a similar strategy may not be feasible in democratic countries. However, experiences during the MERS-related coronavirus epidemic in Taiwan proved that it was also possible to contain the epidemic by early intervention and community participation. The results of our study proved that there was 3-5 weeks for the SARS-CoV-2 epidemic to transit through the subexponential phase. If there had been an effective public health response in time, the exponential transmission could have been averted. We showed earlier that unplanned lockdown would enhance the exposure to the infection due to panic shopping and overcrowding in bus and train stations [35].

In India, the opportunity to favorably contain the exponential transmission was missed due to inadequate testing and contact tracing, especially in the overcrowded metro cities and urban settings. Exposures in religious meetings and marketplaces resulted in several epidemic clusters in Chennai City. Now, the three major cities Mumbai, Chennai, and Ahmadabad contribute about 58% of the total cases in India, and there are claims that there are only clusters of transmission in India. The results of our study show that the country needs an exclusive containment strategy in urban areas, in particular in metropolitan cities.

The modeling outcome also forecasts the probability of the infection in the metro zone when public transports are opened up after the lockdown. The long hours of travelling in a congested metro zone enhances the exposure, even if there is a single infected person in the closed environment. Train travel appears to be safer, although the travelling time is the same for the longest travel in the constructed study area due to the architecture of the train compartment that provides more air volume for the travelers. Our model did not include random contact with the infected person to estimate the probability of the infections and the resultant secondary infection. It is desirable to apply network modeling for precise estimation of the secondary infections.

## Abbreviations

COVID-19: coronavirus disease
MERS: Middle East respiratory syndrome
MTC: Metropolitan Transport Corporation
R_A_: secondary infections
R_0_: reproductive number
SARS: severe acute respiratory syndrome
SARS-CoV-2: severe acute respiratory syndrome coronavirus 2
